# The Angiotensin Converting Enzyme Insertion/Deletion Polymorphism Modifies Exercise-Induced Muscle Metabolism

**DOI:** 10.1371/journal.pone.0149046

**Published:** 2016-03-16

**Authors:** David Vaughan, Michael Brogioli, Thomas Maier, Andy White, Sarah Waldron, Jörn Rittweger, Marco Toigo, Jessica Wettstein, Endre Laczko, Martin Flück

**Affiliations:** 1 Institute for Biomedical Research into Human Movement and Health, Manchester Metropolitan University, Manchester, United Kingdom; 2 Institute of Human Movement Sciences and Sport, ETH Zurich, Zurich, Switzerland; 3 Chester Marathon, Chester, United Kingdom; 4 University Hospital Balgrist, University of Zurich, Zurich, Switzerland; 5 Functional Genomics Center Zürich, UZH/ETHZ, Zurich, Switzerland; National Research Council of Italy, ITALY

## Abstract

**Objective:**

A silencer region (I-allele) within intron 16 of the gene for the regulator of vascular perfusion, angiotensin-converting enzyme (ACE), is implicated in phenotypic variation of aerobic fitness and the development of type II diabetes. We hypothesised that the reportedly lower aerobic performance in non-carriers compared to carriers of the ACE I-allele, i.e. ACE-DD vs. ACE-ID/ACE-II genotype, is associated with alterations in activity-induced glucose metabolism and capillarisation in exercise muscle.

**Methods:**

Fifty-three, not-specifically trained Caucasian men carried out a one-legged bout of cycling exercise to exhaustion and/or participated in a marathon, the aim being to identify and validate genotype effects on exercise metabolism. Respiratory exchange ratio (RER), serum glucose and lipid concentration, glycogen, and metabolite content in *vastus lateralis* muscle based on ultra-performance lipid chromatography-mass spectrometry (UPLC-MS), were assessed before and after the cycling exercise in thirty-three participants. Serum metabolites were measured in forty subjects that completed the marathon. Genotype effects were assessed post-hoc.

**Results:**

Cycling exercise reduced muscle glycogen concentration and this tended to be affected by the ACE I-allele (p = 0.09). The ACE-DD genotype showed a lower maximal RER and a selective increase in serum glucose concentration after exercise compared to ACE-ID and ACE-II genotypes (+24% vs. +2% and –3%, respectively). Major metabolites of mitochondrial metabolism (i.e. phosphoenol pyruvate, nicotinamide adenine dinucleotide phosphate, L-Aspartic acid, glutathione) were selectively affected in *vastus lateralis* muscle by exercise in the ACE-DD genotype. Capillary-to-fibre ratio was 24%-lower in the ACE-DD genotype. Individuals with the ACE-DD genotype demonstrated an abnormal increase in serum glucose to 7.7 mM after the marathon.

**Conclusion:**

The observations imply a genetically modulated role for ACE in control of glucose import and oxidation in working skeletal muscle. ACE-DD genotypes thereby transit into a pre-diabetic state with exhaustive exercise, which relates to a lowered muscle capillarisation, and deregulation of mitochondria-associated metabolism.

## Introduction

Oxidation of blood-derived substrates in contracting muscle provides the larger portion of energy required during endurance work [1,2,3]. This relies on a series of steps between the hepatic release of glucose and lipid into the blood stream and the metabolization of these substrates, along with amino acids, in working muscle [4]. Perfusion of contracting muscle fibres is the necessary prerequisite for the delivery of substrates to working skeletal muscle [5,6]. Perfusion capacity in exercising muscle is largely set by the number and density of capillaries associated with muscle fibres [7] and the regulation of microcirculation through effects of endothelial shear stress and hormones on vascular tone [5,8].

In this regard an overriding of the vasoconstriction in resting muscle can permit the early increase in muscle perfusion with the onset of contractions [5,9]. In this respect the inhibition of the vasoconstrictive action of angiotensin 2 possibly exerts a quantitatively important contribution to the degree of muscle perfusion [10,11,12]. Intriguingly, a distinct gene polymorphism, i.e. ACE-I/D, for the upstream regulator of angiotensin 2, angiotensin converting enzyme (ACE), is associated with endurance performance through the regulation of ACE activity [13,14]. The ACE-I/D polymorphism is characterised by either the presence (I-allele) or the absence (D-allele) of a silencer sequence in intron 16 of the ACE gene [15]. Presence of the I-allele thus results in lower ACE activity in blood serum and in reduced expression of the ACE gene transcript and of the ACE protein in skeletal muscle, potentially reducing the capacity for angiotensin 2 production [14,16]. Both alleles of the ACE-I/D polymorphism are frequent in Caucasian populations [17]. The phenotypic differences regarding exercise performance between genotypes of the ACE-I/D polymorphism involve a central cardiopulmonary effect [18] and an altered response of left-ventricular mass in response to physical training [19]. Peripheral components possibly also contribute to the superior endurance performance in subjects with the ACE I-allele. This is indicated by our recent observation of elevated capillarisation in subjects with the ACE I-allele and superior increases in the local components of aerobic substrate metabolism with endurance training, i.e. the volume density of subsarcolemmal mitochondria and intramyocellular lipids [16]. These findings relate to the increase in serum angiotensin 2 levels after intense exercise [10,20] and an increased glucose uptake in contracting muscle after angiotensin 2 infusion [21]. Collectively, the findings highlight an important role of the ACE system in the regulation of glucose metabolism in muscle with exercise. This view is in accordance with the higher risk of ACE-DD genotypes to develop type II diabetes, being characterised by high serum glucose levels [19,22].

The significance of phenotypic variation due to the ACE I-allele on the acute angiotensin 2-related response of muscle metabolism to endurance exercise is yet to be established. We reasoned that differences in metabolic reactions in working muscle should exist between carriers and non-carriers of the ACE I-allele and would relate to a modulated muscle capillarisation and exercise response of angiotensin 2 levels in serum. Specifically, we hypothesised that muscle capillarisation would be diminished in participants with ACE-DD genotype and result in a reduced glucose import and metabolism in muscle with exercise; therefore partially explaining the reported lower endurance performance in this genotype [13,14]. In order to expose the influence of the ACE-I/D polymorphism on exercise-induced metabolism we chose to challenge metabolic control with a one-legged, rather than a two-legged, bout of exhaustive cycling exercise in the fasted state and validate the identified metabolic effect with an exhaustive type of running exercise (i.e. a marathon run).

## Materials and Methods

### Experimental design

Fifty-three male participants were recruited from White British men of the Greater Manchester Area via local newspapers and contacts with sports clubs and the organiser of the Chester Marathon. Exclusion criteria were smoking, long-term ill-health and an age under 18 years or over 40 years, and a relative $\dot{V}{\text{O}}_{2\text{max}}$ below 40 ml O_2_/min/kg or above 60 ml O_2_/min/kg (as determined post-hoc). Participants were asked to carry out lab-based tests on the metabolic response to one-legged exercise and/or to participate in a field test consisting of the Chester Marathon. Lab-based test were carried out during two visits, to estimate aerobic capacity and test the metabolic response to one-legged exercise. Thirty-one subjects (group 1) volunteered for the lab-based tests. Forty subjects (group 2) undertook the field test of the Chester Marathon (S1 Fig). Eighteen subjects participated in the lab-based tests and the marathon (intersection group). The ACE-I/D polymorphism was determined in a double blind manner and assessed post-hoc for its influence only after the physiological and biochemical measurements had been performed. The Ethics committee of Manchester Metropolitan University specifically approved this study. The investigation was conducted according to the principles expressed in the Declaration of Helsinki and according to published guidelines [23,24]. Informed consent, written and oral, was obtained from the participants.

### Lab-based tests

Subjects reported to the laboratory during two visits. During the first visit subjects received an explanation of the study intent and gave their agreement to participate. Subsequently anthropometry was assessed, an ergospirometry test was carried out on a stationary cycle ergometer and a mucosal swaps was collected with a cotton ear bud. During the second visit subjects carried out an exhaustive bout of one-legged endurance exercise on the stationary cycle ergometer at a performance-matched intensity to assess metabolic reactions in serum at the cessation of exercise and in skeletal muscle 30 min after the exercise.

#### Anthropometry

Age, body weight and height were measured and the BMI was calculated during an initial visit. Then subjects completed a lifestyle questionnaire composed of 31 questions as modified from a previous short-form 36 [25].

### Ergospirometry

Measurements of aerobic performance (i.e. $\dot{V}{\text{O}}_{2\text{max}}$ and maximum aerobic power output, *P*_max_) were performed during an incremental endurance test with both legs on a cycle ergometer (Ergometrics Ergoline 800, Jaeger, Bitz, Germany) in an air-conditioned room at 23°C. The measurements were carried out through a mouth-piece with a breath-by-breath technique using a stationary cardiopulmonary exercise testing device (MetaLyser® 3B, Cortex, Leipzig, Germany). The saddle length was adjusted to a position where the knee was extended at an approximately 175° angle when subjects were seated with the shoe heel placed on the pedal. The test started with 2 min of baseline recording followed by 3 min warm-up at 80 W and 80 rpm. Intensity was increased by 25 W every minute until exhaustion. An intensity level was considered achieved when 80 rpm were held for at least 50 s. Respiration was followed into a cool-down phase of 3 min at 80 W and 80 rpm followed by 2 min of rest. Test results were recorded at 3 seconds interval with the MetaSoft® software (Cortex, Leipzig, Germany) and analysed offline with the method ‘maximal oxygen uptake’ for absolute and specific, i.e. body mass related, $\dot{V}{\text{O}}_{2\text{max}}$ and RER following the exercise. $\dot{V}{\text{O}}_{2\text{max}}$ was identified based on the criteria that $\dot{V}{\text{O}}_{2}$ reached a plateau of a steady maximal value under the imposed high workload, when RER was above 1.05, and before $\dot{V}{\text{O}}_{2}$ fell off because the pedal rate fell consistently below 70 rpm despite verbal encouragement (for a review see [26]). The $\dot{V}{\text{O}}_{2}$ values at the plateau varied within 1% of the average values and the plateau was maintained on average over 26 seconds. $\dot{V}{\text{O}}_{2\text{max}}$ was determined as the highest mean of $\dot{V}{\text{O}}_{2}$ values averaged over a period of 30 seconds in the plateau phase. In the case a $\dot{V}{\text{O}}_{2}$ plateau did not manifest during the test, the ergospirometry was repeated on a subsequent day.

### One-legged endurance exercise

Subjects reported after an overnight fast and 2 days of reduced physical activity to the laboratory. A resting biopsy was collected under anaesthesia from the *vastus lateralis* muscle of the non-dominant leg. A 5-ml blood sample was drawn from the Cephalic vein into a tube sprayed with dry EDTA (K2E BD Vacutainer^®^, Belliver Industrial Estate, Phymouth, UK) and placed on ice. A 2-mL aliquot was rapidly processed as described below under the paragraph ‘*quantification of serum angiotensin 2 concentration*’. The residual blood serum was subjected to the measure of metabolites as described under the respective paragraph ‘s*erum metabolites’*.

Subsequently, subjects completed a one-legged exercise test with the dominant leg at a set cadence of 80 rpm on the stationary cycle ergometer (Ergometrics Ergoline 800, Jaeger, Bitz, Germany) where the pedal for the non-dominant leg was taken off. Saddle length was set to the value used for the two-legged exercise. The shoe of the dominant leg was attached to the pedal with duct tape. The other leg rested on the frame in the middle of the ergometer. Subjects initially performed a warm-up at 15% of the predicted 2-legged *P*_max_ which was followed by 25 min of exercise at 30% of the 2-legged *P*_max_ before the set intensity was ramped up in 10 W increments per minute until exhaustion. A 3-min cool-down phase at 15% of the calculated 2-legged *P*_max_ was allowed at the end of exercise. $\dot{V}{\text{O}}_{2}$, $\dot{V}{\text{CO}}_{2}$ and ventilation were monitored with the MetaLyser® 3B system (Cortex, Leipzig, Germany) and $\dot{V}{\text{O}}_{2\text{max}}$ and maximal RER was determined.

### Field test

Participants registered for the Chester Marathon in Northwest England, which took place on 9^th^ October 2011. The race time was monitored with a chip-based system at the start, mid and finish line of the race. Capillary blood was collected from each finisher within 5–10 min after completing the marathon: First the fingertip was cleaned and sterilised with an ethanol wipe and a small superficial incision was made with the help of a 2.25 mm depth sterile blood lancet (Safety Lancet; HemoCue®; Ängelholm; Sweden). After a blood droplet was formed, about 30 μL of blood were collected with heparinized capillary plastic tubes and subjected to the analysis of glucose and triglyceride concentration as described in the section ‘s*erum metabolites’*. A mucosal swab was collected to assess the ACE-I/D genotype. The name, race time and number was written down and verified later with the race director.

### Genotyping

The collected mucosal swab was frozen at –20°C in a sealed 15 ml tube (Sarstedt; Nümbrecht; Germany). DNA was extracted after thawing with 800 μL of methanol. The solution was air dried, frozen over-night at –80°C and resuspended in 100 μL of sterile water under heating to 65°C. DNA was recovered in the supernatant after a centrifugation step (5000 g, 2 min, room temperature) and stored at –20°C. Genotyping for the ACE-I/D polymorphism was carried out in a double blind manner. Towards this end, sample codes were blinded by a second investigator by sticking a label with random, but unique, four letter code on top. The code was handed to a third investigator unrelated to the study. Subsequently the DNA samples were subjected together with mock and camouflage samples to a polymerase chain reaction to type the ACE-I/D polymorphism according to the protocol as described in [16]. The genotyping results were decoded through the involvement of the third investigator once the functional test and metabolic measures had been completed.

### Biopsy

An experienced physician collected the muscle biopsies during the second lab-based visit; one at rest from the non-exercising leg, and one 30 min after one-legged exercise from the dominant leg, which performed to exercise. The samples were taken from the vastus lateralis muscle, at the point of maximal thickness. The overlying skin was shaved and sterilised (Videne Antiseptic Solution, Ecolab, Saint Paul, MN USA). A sterile drape from a wound care pack (Premier, Shermond Bunzel Retail & HealthCare Supplies Limited, Enfiled, Middlesex, UK) ensured sterile conditions. For local anaesthesia, 1 ml 2% Lidocaine was injected subcutaneously. Within 5 min a 0.5-mm incision was made with a scalpel and muscle sample was extracted using a biopsy needle (TSK Acecut 11G, Emergo Europe, The Hague, The Netherlands) and immediately processes by an investigator. Firm pressure was applied to the biopsy site until the bleeding stopped. The wound was then closed (Steri-Strip, 3M Health Care, Germany) and dressed (Mepore Ultra, Molnlycke Healthcare, Sweden). Subjects were discharged with a pressure bandage for the first 4 h after the biopsy sample to reduce any further bleeding.

Biopsy samples were rapidly frozen in liquid nitrogen while shaking the sample. Pre-exercise samples were cut into two pieces before being frozen; one being mounted with Tissue-Tek^®^ O.C.T. TM Compound (Weckert Labortechnik, Kitzingen, Germany) on cork for histological analysis before freezing. Samples were stored airtight in a 2-mL tube (Eppendorf) until further processing.

### Measurements of capillarisation

Capillaries were detected and analysed essentially as described [27]. In brief, pre-exercise biopsies were mounted, and cryosections prepared at 14-μm thickness under a cutting angle being perpendicular to the major axis of muscle fibres. Capillaries were detected based on a lectin antibody and the section recorded at a 10x magnification with an Axiocam MRc camera being operated by a Axioskop 2 mot plus stage (Carl Zeiss, Oberkochen, Germany). Areas of the section corresponding to 0.15 mm^2^ where fibres were cut perpendicular and where no holes or other irregularities were present were selected. The areas were processed with the Image J 1.6.0_33 J software (http://imagej.nih.gov/ij) according to the published settings [27] to determine the number of capillaries per square millimetre (capillary density) and the capillary-to-fibre ratio. The values of at least 24 representative fibres per subject were analysed.

### Muscle fibre cross sectional area

Mean cross sectional area was determined from cryosections after immunohistochmical staining with fast type myosin heavy chain antibody (M4276, Sigma Chemicals, Buchs, Switzerland) and quantifying the area of stained (fast type) and non-stained (slow type) muscle fibres in a given microscopic field was quantified using image J 1.6.0_33 J (http://imagej.nih.gov/ij) as described [28]. On average 133 fibres were measured per cross-section.

### Quantification of serum angiotensin 2 concentration

Angiotensin 2 levels were quantified with a validated commercial angiotensin II enzyme-linked immunoabsorbent assay (SPIBio Bertin Pharma, Montigny le Bretonneux, France) essentially as described [10].

### Serum metabolites

30 μL of capillary blood was used to measure the main metabolic substrates (glucose, triglycerides, cholesterol, high-density lipoprotein (HDL) cholesterol and/or ketones) using a portable whole blood test system (CardioCheck®, Polymer Technology Systems; Indianapolis, IN, USA). Glucose concentration was measured in the first minute after collection. The serum concentration of low-density lipoprotein (LDL) and very low-density lipoprotein cholesterol (VLDL-C) was calculated as described [29]. The coefficient of variation for repeated measurements was 2.6% for glucose and 3.9% for triglycerides, respectively.

### Muscle metabolites

Metabolite profiles of biopsy samples, collected before and 30-min after one leg exercise based on established protocols [30,31], were analysed using ultrahigh performance liquid chromatography–tandem mass spectrometry (UPLC–MS). In brief, 5 mg muscle tissue was extracted in cold MetOH: MTBE: H20 = 360: 1200: 348 using a full glass Potter type homogeniser. 10 μL of a 50-μM solution of each LysoPC (17:0) (Avanti Polar Lipids) and ^13^C-Sorbitol (Sigma) was added as internal standards. The upper (non-polar) phase with lipids and the bottom (polar phase) with metabolites were separated by a centrifugation step (5min at 1000g, at 4°C), separately collected and stored at –30°C. Prior to analysis the metabolite extracts were dried down under a steam of nitrogen and reconstituted in 100 μL of 50mM ammoniumacetate in acetonitrile–water 9:1 (v/v). Metabolites were separated on nanoAquity UPLC (Waters) equipped with a BEH-Amide capillary column (200 μm x 150 mm, 1.7 μm particle size, Waters), applying a gradient of 0.5 μM ammoniumacetate in acetonitril (A) and 0.5 μM ammoniumacetate in water (B) from 90% A to 50% A. The injection volume was 1 μL. The UPLC was coupled to Q Exactive™ Hybrid Quadrupole-Orbitrap Mass Spectrometer (Thermo Fisher Scientific, Reinach, Switzerland) by a nanoESI source. MS data was acquired using negative polarization and all ion fragmentation (AIF) over a mass range of 80 to 1200 m/z at a resolution of 70’000 (MS) and 25’000 (MSMS). All extractions steps were carried out in dichlormethane-washed Duran glassware. All solvents used were of quality HPLC grade (Chromasolv, Sigma-Aldrich, Buchs, Switzerland). Metabolite data sets were evaluated with Progenesis QI software (Nonlinear Dynamics), which aligns the ion intensity maps based on a reference data set, followed by a peaks picking on an aggregated ion intensity map. Detected ions were identified based on accurate mass, detected adduct patterns and isotope patterns by comparing with entries in the Human Metabolome Data Base (HMDB). A mass accuracy tolerance of 5 mDa was set for the searches. Fragmentation patterns were not considered for the identifications of metabolites. All biological samples were analysed in triplicate and quality controls were run on individual and mixed samples to determine technical accuracy. This analysis was carried out for 20 selected compounds (amino acids, nucleotides, and metabolic intermediates) in mixed samples using Quan Browser (Xcalibur, Thermo Fisher Scientific) and 61 further abundant ions using Progenesis QI software (Nonlinear Dynamics). The coefficients of variation for biological and technical replicas of the 20 compounds in mixing experiments demonstrated values near or below 20%. The average coefficient of variation for the repeated measure of ion intensities for the abundant ions was 19.6% for technical replicas and 23.2% for biological replicas. The correlation coefficient (r) between signal intensities from biological replicas was 0.72.

Glycogen was measured relative to the total amount of muscle protein. In brief, cryosections (25 μm) were prepared from muscle biopsies and the section volume estimated from microscopic measures of the cross-sectional area and the height of the sectioned tissue. An approximate of 1 mm^3^ tissue was homogenised in 100 μl of a PBS/inhibitor-cocktail [1 ml PBS + 9 ml dH_2_O + 1 complete Mini, EDTA-free tablet (Sigma Aldrich, Buchs, Switzerland) in a 1.5 ml Eppendorf tube by using a steel pistil (Behrens-Labortechnik, Germany). Total protein content was assessed in 3 μl homogenate against a BSA standard using the Pierce BCA Protein Assay Kit (Thermo SCIENTIFIC, Town, USA) and quantified at 562 nm on a 96-well plate with a Synergy HT spectrometer (BioTek Instruments Inc., Vermont USA). Glycogen was measured on 20 μl muscle homogenate against a glycogen standard with the Assay Kit (abcam, Cambridge, UK) according to the instructions. The reaction was developed at room temperature in the dark in a 96-well plate in the dark. Signal was detected at 564 nm using a Synergy HT spectrometer (BioTek, Lucerne, Switzerland). The coefficient of variation for repeated measurements of the standard curve on different days was 3.1% for BSA-based measures of protein content and 0.1% for glycogen, respectively.

### Statistics

Genotype differences prior to exercise for non-repeated factors (age, body mass, height, BMI, body mass-specific $\dot{V}{\text{O}}_{2\text{max}}$, $\dot{V}{\text{O}}_{2\text{max}}$, *P*_max_) were assessed with a one-way ANOVA for the factor genotype (ACE-DD, ACE-ID, ACE-II) using Statistica software (Statistica, StatSoft, Tulsa, USA). Statistical significance of post vs. pre exercise alterations for the assessed parameters (RER, serum metabolites, muscle glycogen concentration) was assessed with a repeated ANOVA for the repeated factor time (pre exercise, post exercise) and the factor genotype (ACE-DD, ACE-ID, ACE-II) using Statistica software (Statistica, StatSoft, Tulsa, USA). Post-hoc tests of Fisher were used to localise the statistical significance of differences between genotypes (Statistica). Significance was accepted at p-value < 0.05; trends were declared at p < 0.10. Results are presented as median ± standard error (SE). Hardy-Weinberg equilibrium was assessed by submitting the numbers to the online tool: http://www.had2know.com/academics/hardy-weinberg-equilibrium-calculator-2-alleles.html. Linear relationships were assessed based on Pearson correlations and considered to be significant if p < 0.05 (Statistica, StatSoft, Tulsa, USA).

For the assessment of ion abundance, the metabolite data set was limited to compounds being detected in all analysed samples. For each sample, raw signals of each compound were normalised to the total signal of detected compounds for that sample; revealing the relative fraction of ion abundance for the respective compound. Changes in compounds were assessed based on permutations of *t*-tests using statistical analysis of microarrays (SAM) [32]. A paired class design was applied to identify post vs. pre changes in metabolites for each genotype (i.e. ACE-DD, ACE-ID and ACE-II). Genotype differences in fold changes with exercise were assessed with a SAM based on an unpaired design. A false discovery rate (FDR) of 5% was deemed significant. Output was exported into Microsoft-Excel (Microsoft Office for Windows, Kildare, Ireland) for table assembly and the functional significance of the altered metabolites was assessed based on HMBD entries (http://www.hmdb.ca/metabolites/).

## Results

### Baseline values

Table 1 visualises the characteristics of the 31 subjects, which completed the lab-based tests. Anthropometric variables and parameters of endurance exercise capacity (i.e. $\dot{V}{\text{O}}_{2\text{max}}$ and *P*_max_) did not differ between ACE-I/D genotypes. The observed genotype frequencies were in agreement with Hardy-Weinberg equilibrium (Chi^2^ = 1.807, p = 0.412).

**Table 1:** *Subject characteristics—*Median and SE of anthropometric and performance measures and p-values of the subjects of group 1 which completed the lab-based tests, and the intersection group which performed lab-based tests and the Marathon. ANOVA with post-hoc test of Fisher.

genotype	n	Age	Weight	Height	BMI	mass-specific $\dot{V}\text{O}2\text{max}$	Pmax	$\dot{V}\text{O}2\text{max}$
		[years]	[kg]	[cm]	[kg/m^2^]	[mlO_2_/min/kg]	[Watt]	[mlO_2_/min]
**group 1:**								
ACE-DD	11	25.0 ± 1.6	72.0 ± 2.9	182.0 ± 2.4	22.1 ± 0.6	55.6 ± 2.2	302.5 ± 14.9	3960.4 ± 203.6
ACE-ID	13	31.0 ± 1.4	77.9 ± 2.9	179.0 ± 1.4	23.6 ± 0.9	49.0 ± 2.2	305.0 ± 14.3	3974.4 ± 185.6
ACE-II	7	23.0 ± 2.9	77.5 ± 6.0	184.0 ± 4.1	23.5 ± 1.4	50.1 ± 3.3	365.0 ± 34.4	4435.0 ± 396.5
ALL	31	27.1 ± 1.8	75.9 ± 3.6	181.7 ± 2.4	23.1 ± 0.9	51.5 ± 2.4	323.5 ± 19.1	4142.9 ± 239.6
*p-values*								
DD vs. ID/II		0.06	0.26	0.96	0.16	0.27	0.5	0.89
DD vs. II		0.90	0.47	0.98	0.36	0.69	0.27	0.67
**Intersection group:**								
ACE-DD	3	27.6 ± 3.8	71.3 ± 3.9	179.0 ± 0.7	21.1 ± 1.1	56.0 ± 3.5	305.0 ± 25.0	3930.8 ± 40.6
ACE-ID	11	35.6 ± 2.1	74.0 ± 0.7	178.5 ± 1.3	23.5 ± 0.3	55.0 ± 2.7	318.0 ± 17.5	3900.0 ± 125.6
ACE-II	4	33.9 ± 2.1	71.1 ± 0.7	169.0 ± 1.7	24.5 ± 0.6	55.0 ± 2.3	317.5 ± 2.5	3701.0 ± 111.4
ALL	18	33.4 ± 1.6	73.3 ± 1.5	178.5 ± 2.1	23.5 ± 0.5	55.0 ± 1.5	317.5 ± 12.0	3775.1 ± 81.1
***p-values***								
DD vs. ID/II		0.07	0.26	0.55	0.13	0.43	0.6	0.77
DD vs. II		0.23	0.79	0.04	0.03	0.31	0.67	0.19
1st vs. intersection group:		0.02	0.07	0.18	0.45	0.12	0.64	0.78

### Genotype effects of exhaustive exercise on metabolism

**Fig 1:**
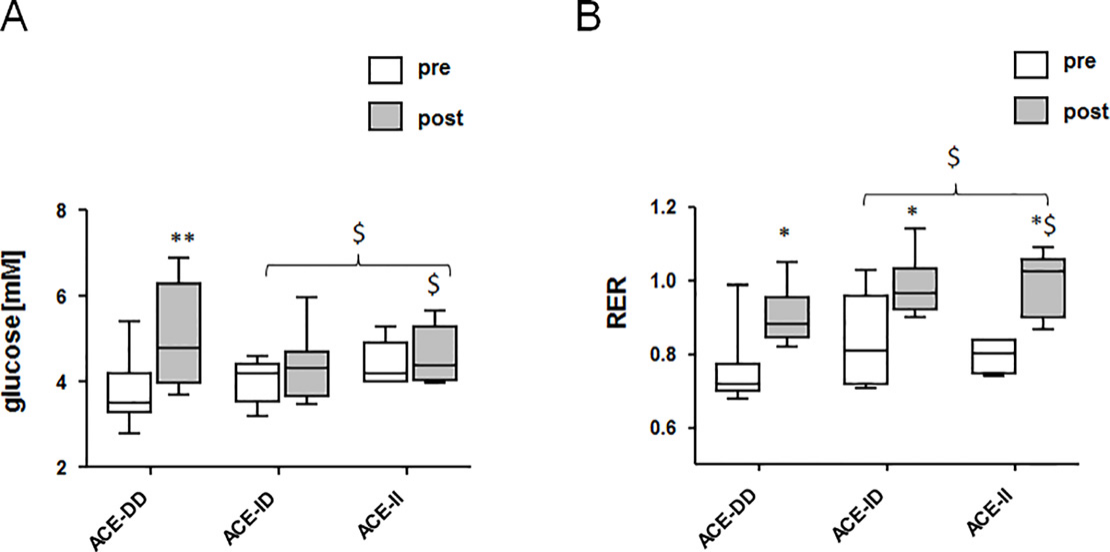
Genotype effects on exercise induced metabolism. Whisker box plots visualizing the median + standard error (box and central line) and minima/maxima (whisker) of serum glucose (A) and RER (B) pre and post the exhaustive one leg exercise for group 1. *, p < 0.05 vs. pre; **, p < 0.001 vs. pre; $, p < 0.05 vs. ACE-DD post exercise (repeated ANOVA with post-hoc test of Fisher). Swung bracket indicates effect for combined ACE-II and ACE-ID genotypes.

One-legged cycling exercise to exhaustion did increase RER (p = 0.011). The maximal values of RER at the end of the exercise were lower in subjects of the ACE-DD genotype, compared to subjects carrying the I-allele (p = 0.040), i.e. ACE-ID or ACE-II genotypes (Fig 1B). The serum concentration of glucose, but not triglyceride, lipoprotein and ketone concentration was increased in ACE-DD genotypes after the exercise and this differed to the response of subjects with the ACE I-allele (S1 Table, Fig 1A).

Serum concentration of the ACE product, angiotensin 2, was higher in ACE-DD genotypes than in ACE-II and ID genotypes before (29.0 ± 3.1 vs. 6.5 ± 2.2 pg/ml, p = 0.01), but not after (24.2 ± 3.1 vs. 28.2 ± 18.8 pg/ml, p = 0.45), the one-legged cycling exercise.

### Genotype effects on muscle metabolism during early recovery from exercise

**Fig 2:**
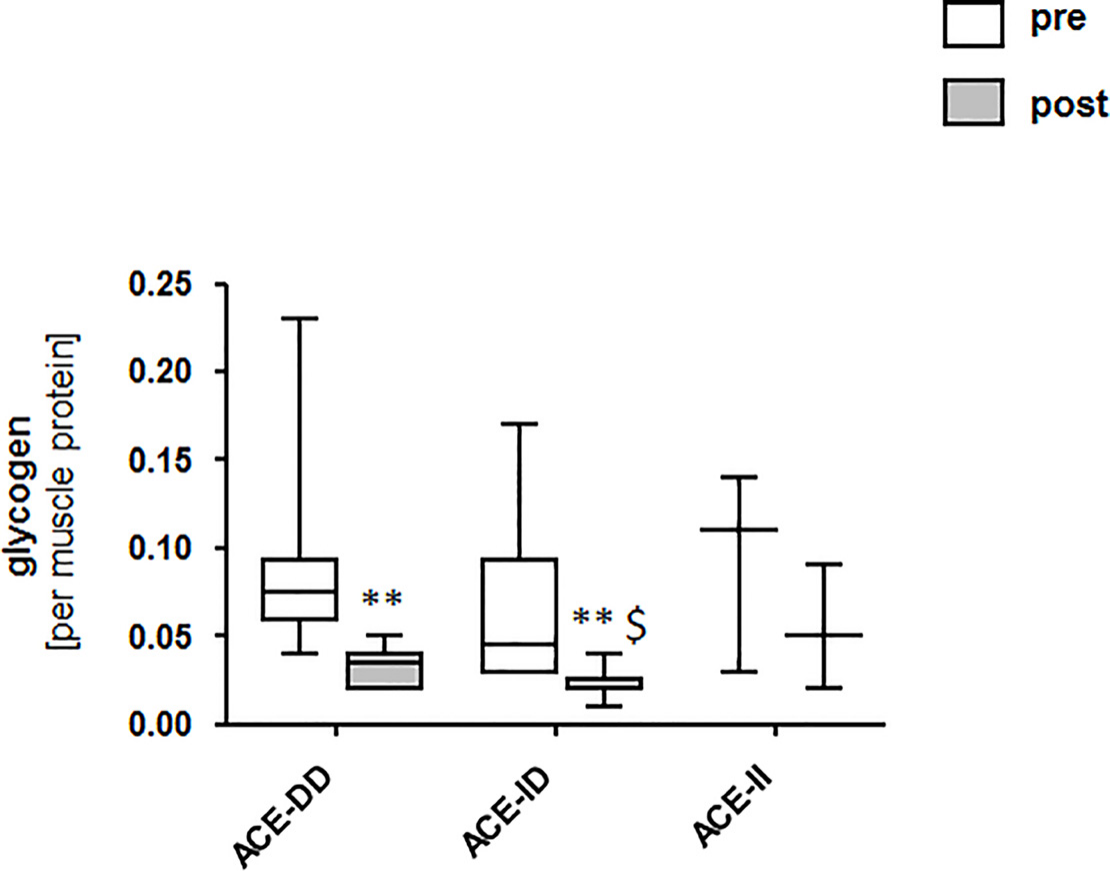
ACE I/D genotype effects on muscle metabolism post exercise. Whisker box plots visualizing the median + standard error (boxes) and minima/maxima (Whiskers) of muscle glycogen concentration pre and post the exhaustive one leg exercise for group 1. *, p < 0.05 vs. pre; **, p < 0.001 vs. pre; $, p < 0.05 vs. ACE-DD post exercise (repeated ANOVA with post-hoc test of Fisher).

We assessed the contribution of muscle metabolism to the suggested difference in glucose consumption with exhaustive endurance exercise. Glycogen content in *vastus lateralis* muscle was reduced 30 min after the one-legged exercise in all subjects (p = 4.5E-5). After the exercise, subjects with the ACE-ID genotype had a lower glycogen concentration than those with the ACE-DD genotype (Fig 2B).

UPLC–MS based methodology detected 924 metabolites in *vastus lateralis* muscle; 18 of which showed altered levels 30 min after exercise. Five of those were mapped to existing data base entries; the major theme being an increase in compounds associated with amino acid metabolism (S2 Table). Conversely, 2059 lipidic compounds were detected and 79 compounds altered their level 30-min after the one-legged exercise. 13 of these were identified based on HMDB entries (S2 Table).

**Fig 3:**
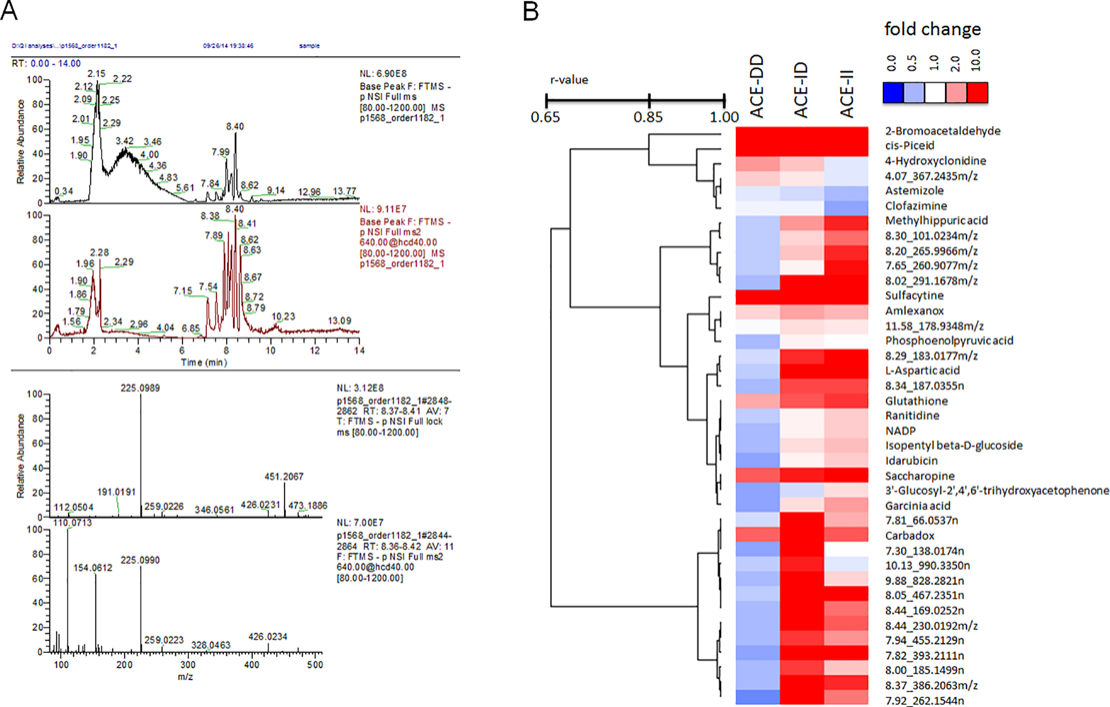
Profiling the ACE I/D genotype dependent changes of the muscle metabolome with exercise. (A) Base peak intensity chromatogram of MS and MSMS data (top two panels) and the corresponding averaged mass spectra over the retention time range 8.37 min to 8.42 min (peak with apex at 8.40 min); likely under the peak at 8.40 min there are 4 major co-eluting compounds with m/z 225.0989 ([M-H]-), 259.0223 ([M-H]-), m/z 451.2067 and 473.1886 (likely 2 adducts of the same compound, i.e. [M-H]- & ([M+Na-H]-) and 426.0231 ([M-H]-). In the MSMS spectra fragments with m/z 110.0713, 154.0612 and 328.0463 and some minor fragments are visible. (B) Heatmap visualising the average fold changes of the 39 compounds demonstrating genotype specific level alterations after the exhaustive bout of one-legged exercise. Post vs. pre-exercise changes are provided in colour coding: red; up; blue, down. Names of the compounds are listed to the right, either by the description corresponding to the respective HMDB entry, or the composite identifier based on UPLC retention times and the m/z-value of the separation by mass spectrometry.

**Fig 4:**
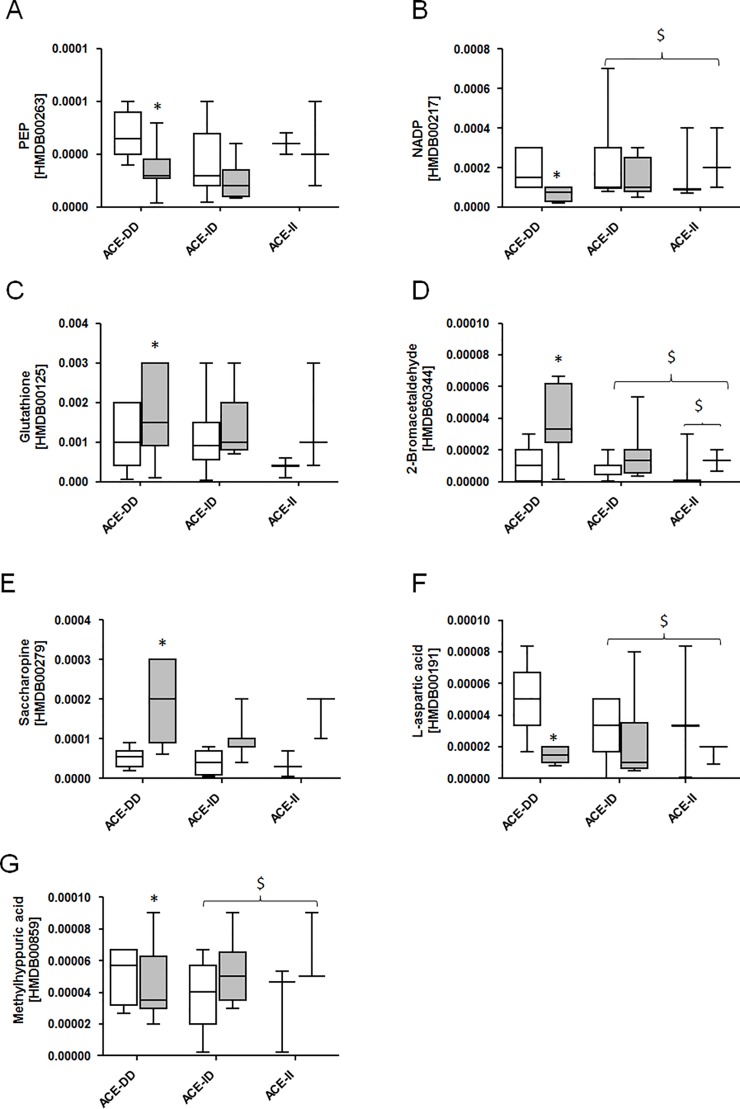
Muscle metabolites after exercise in relation to the ACE I/D genotype. (A-G) Whisker box plots of muscle metabolite levels being associated with pyruvate metabolism (A,B), glutathione metabolism (C,D) and amino acid metabolism (E,F), pre and post exercise per ACE-I/D genotype for group 1. Levels reflect the relative fraction of ion abundance for the respective compound (i.e. metabolite). Respective HMDB entries are provided in brackets. The main function/ ontology of each metabolite is provided in the heading. *, p < 0.05 vs. pre; $, p < 0.05 for fold changes vs. ACE-DD (SAM).

39 compounds demonstrated ACE-I/D genotype dependent alterations after exercise; 19 of which could be mapped to HMDB entries (Fig 3; S3 Table). This comprised important metabolites such as phosphoenol pyruvate (PEP), aspartic acid, glutathione, saccharopine and nicotinamide adenine dinucleotide phosphate (NADP+), all of which were reduced 30 min after the one-legged exercise (Fig 4). Furthermore, ACE-I/D genotype specific alterations of 12 metabolites related to chemical foods and pharmaceuticals were noted (S3 Table). The mainly affected pathways concerned pyruvate metabolism, amino acid metabolism, glutathione redox reactions and glycolysis and gluconeogenesis.

### Genotype dependent muscle substrate pathways

**Fig 5:**
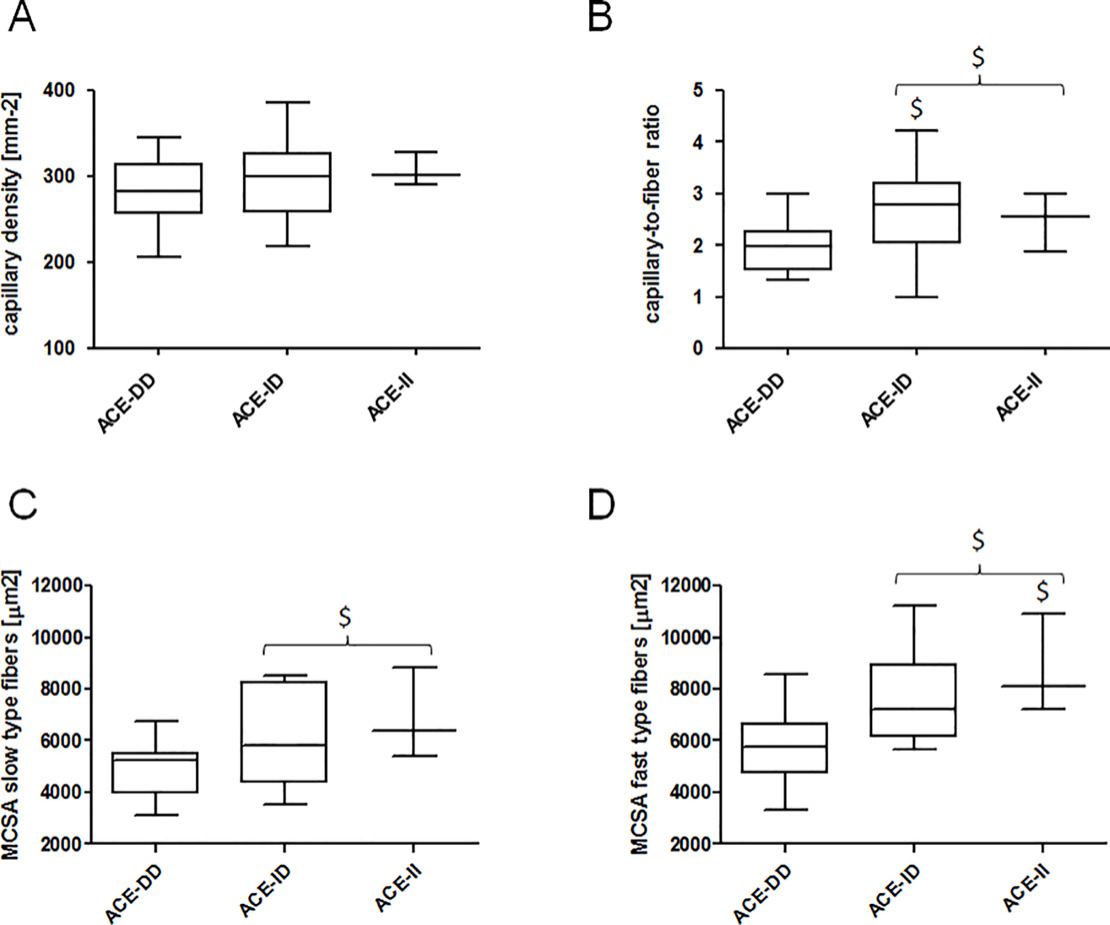
Muscle composition in relation to the ACE I/D genotype. Whisker plots of capillary density (A), capillary-to-fibre ratio (B), and mean cross-sectional area of slow (C) and fast fibre types (D) in *vastus lateralis* muscle in relation to ACE-I/D genotypes for group 1. $, p < 0.05 vs. ACE-DD (ANOVA with post-hoc test of Fisher). Swung bracket indicates effect for combined ACE-II and ACE-ID genotypes.

We assessed the extent to which altered metabolites relate to capillarisation in the recruited skeletal muscle. Capillary-to–fibre ratio in *vastus lateralis* muscle of ACE-II and ID genotypes was elevated compared to ACE-DD genotypes (Fig 5). Fast and slow type muscle fibres demonstrated 39% and 12%, respectively, larger cross-sectional area in ACE-II and ID than ACE-DD genotypes (Fig 5).

### Genotype effect of an exhaustive type of running exercise

**Fig 6:**
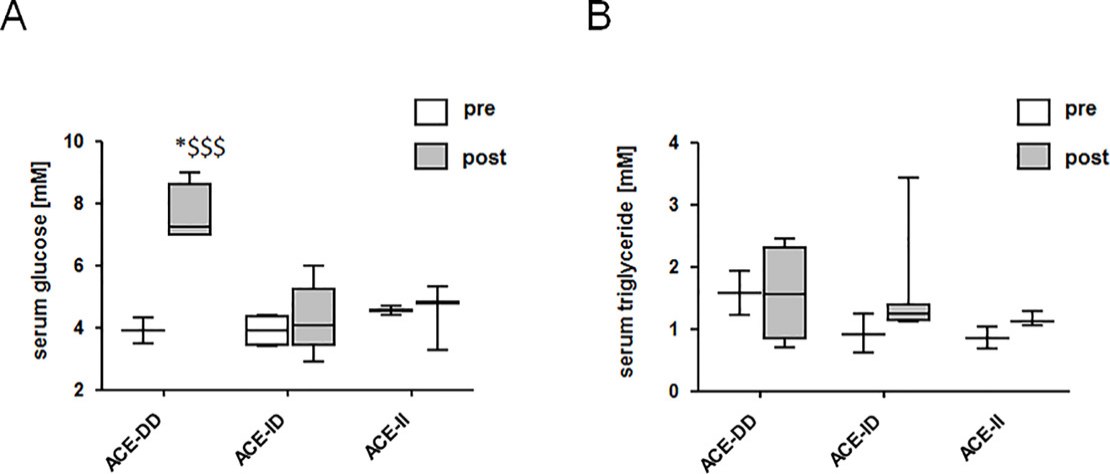
ACE I/D-related alterations in serum after the marathon. Whisker box plots of glucose (A) and triglyceride concentration (B) before and after the Chester Marathon for each ACE-I/D genotype for group 2. *, p < 0.05 vs. pre; $ $ $, p < 0.0001 vs. ACE-DD post exercise (repeated ANOVA with post-hoc test of Fisher).

Forty subjects of group 2 were followed during the Chester Marathon to test whether ACE-I/D genotype dependent alterations in glucose metabolism with cycling exercise can be confirmed. Serum glucose, but not triglyceride, concentration was 80% higher after the marathon in the ACE-DD genotypes than the ACE-II and ID genotypes (Fig 6). No difference was noted for the mean race time between ACE-DD genotypes and ACE-II and ID genotypes (214 vs. 213 min, p = 0.96, unpaired *t*-Test). Five participants with ACE-II or ID genotype, but no subject with the ACE-DD genotype, completed the marathon in less than 3 h.

### Relationships between metabolic parameters

A number of linear relationships were identified between the metabolic parameters and performance in the field and lab test. For the 31 subjects of group 1, which completed the lab-test (Table 1), this concerned the trend for a correlation between average RER and the changes in serum glucose with the one-legged endurance exercise (r = 0.70; p = 0.08) and the correlation between the changes in RER after the exercise and muscle capillary density (r = 0.65).

For the 18 subjects of the intersection group 1, which completed the marathon and attended the lab-based tests (Table 1), this concerned correlations between the time to complete the marathon and capillary density (r = –0.61), $\dot{V}{\text{O}}_{2\text{max}}$ (r = –0.78), BMI (r = 0.69), and glucose concentration prior to the marathon (r = –0.66).

## Discussion

The ACE-I/D polymorphism is a genuine example for genetical effects on endurance performance [33,34]. It has been pointed out before that Caucasian subjects carrying the ACE-I allele show better trainability of endurance performance [35]. Our recent investigation with Caucasian subjects suggested that this has a muscle element [16]. Specifically this involved a higher capillary volume density and an accentuated increase in the volume density of myocellular structures (i.e. subsarcolemmal mitochondria and intramyocellular lipids) that set fatigue resistance with cycling-type endurance training in subjects with the ACE-I-allele [16]. The comparison of the response to exhaustive cycling exercise identified that ACE-DD genotypes showed compared to ACE-ID and ACE-II genotypes a pronounced perturbation in serum glucose concentration, concomitantly with a reduced RER and modified abundance of critical muscle metabolites in knee extensor muscles.

### Model considerations and limitation

Forward to testing the hypothesis on a capillary-related reduction in glucose metabolism during exercise in ACE-DD genotypes we assessed the metabolic response to a bout of one-legged cycling exercise and verified selected markers of metabolism after the exhaustive physical effort of a marathon race. Our rationale for assessing the response to a bout of one-legged cycling exercise was based on the reported central limitations for substrate supply to peripheral muscle, when working at maximal aerobic performance [36] and the central cardiopulmonary effect of the ACE-I/D polymorphism [18]. The intention was to limit ACE-I/D polymorphism related central effects.

Our investigation into the metabolic response was carried out with two partially overlapping groups of participants. Potentially this may have confounded the identification of genotype differences although, except for age, no significant difference existed for the measured anthropometric and physiological variables between group 1 and the intersection group (Table 1). In order to identify muscle metabolites that demonstrate ACE-I/D genotype dependent alterations with exhaustive endurance exercise we opted to use an explorative UPLC-MS approach. This allowed to characterize compounds over a large mass range but had the shortcoming of a reduced precision. The observed coefficients of variation were however within the accepted range for the selected application [37], and we find that a majority of identified compounds passed the threshold for statistical significance at a false discovery rate adjusted q-value below 0.1% (S2 Table and S3 Table). The relevance and robustness of our approach is further supported by the observation that the identified genotype difference in serum alterations of glucose after one-legged exercise was confirmed after the exhaustive type of running exercise as presented by the completion of a marathon.

A number of factors potentially explain the suggested difference in glucose handling between carriers and non-carriers of the ACE I-allele. In this regard the identified changes in muscle metabolites during the early recovery from exercise (Fig 4, S3 Table) provide a signpost of the underlying metabolic processes. The identified ACE-I/D genotype dependent metabolites 30 min post exercises were associated with the metabolism of pyruvate (PEP, NADP), glucose and amino acids (saccharopine, L-aspartic acid, methylhyppuric acid) as well as of glutathione (glutathione, 2-bromacetaldehyde; Fig 4; S3 Table). These metabolic processes are central to metabolic functioning thus possibly suggesting their contribution to differences in substrate utilization between ACE-ID genotypes. In this regard, the lowered abundance of the reduction equivalent NADP and the glycolytic intermediate PEP would be in line with a reduced glycolytic energy production; leading to an attenuated decrease in glycogen content in ACE-DD genotypes (Fig 2A). Support for the ACE I/D polymorphism-dependent modification of glucose metabolism in working muscle was presented by a reduced RER during the exhaustive exercise in ACE-DD genotypes (Figs 1 and 2). In this regard the concomitant reduction of methylhippuric acid is of interest as this is a marker of a deregulated ß-oxidation of fatty acids. Notably, the lowered abundance of L-Aspartic acid and the concomitant increase in saccharopine (a degradation product of lysine) are suggestive for an elevation of amino-acid degradation in the exercised muscle of ACE-DD genotypes after the exhaustive one-legged exercise.

In our experiments we identified that negative linear relationships existed for three metabolism-related parameters and the time to complete the marathon race, i.e. glucose concentration prior to the race, capillary density and $\dot{V}{\text{O}}_{2\text{max}}$. None of these factors demonstrated a significant effect of the ACE I/D genotype. We interpret this observation as to reflect the role of the aerobic oxidation of glucose for performance in the exhaustive type of exercise [38]. Psychological factors aside, these findings suggest that exercise-induced changes in aerobic metabolism and metabolic stores, rather than ACE-modulated parameters did relate to race performance in the subjects under investigation.

**Fig 7:**
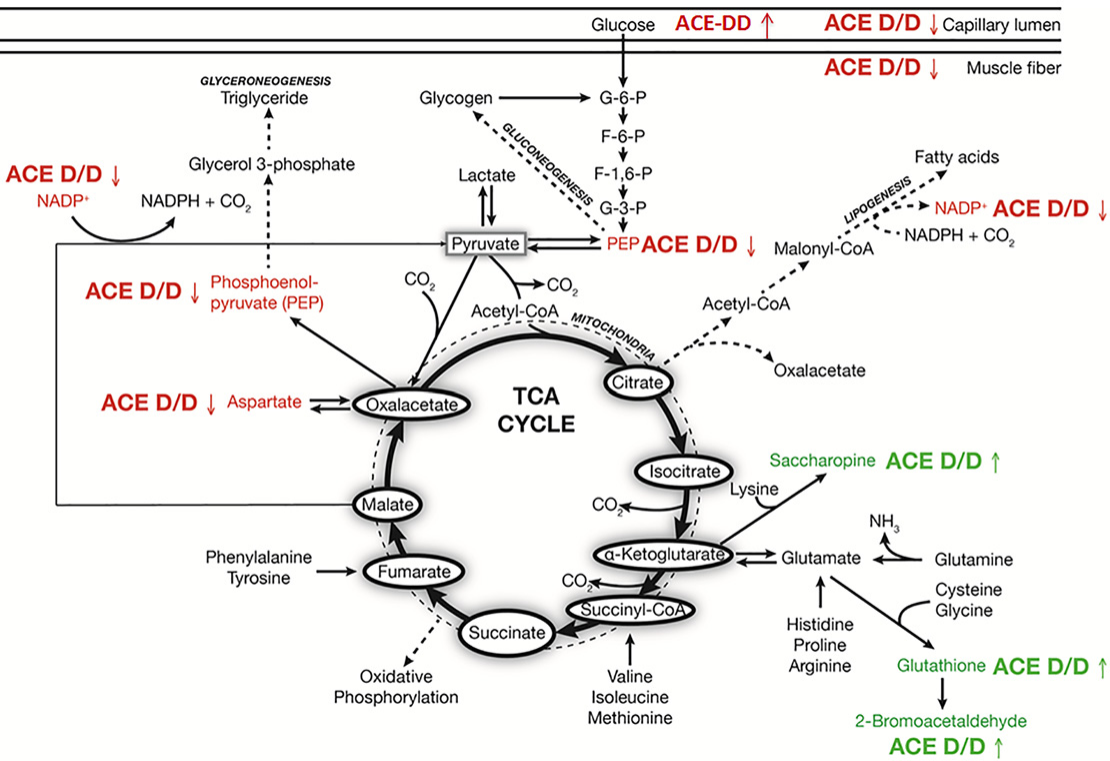
Composite panel of the metabolic features in knee extensor muscle, which demonstrate ACE I/D dependent regulation. Arrows indicate the direction of changes of metabolites in ACE-DD genotype during recovery from exhaustive exercise and associated structural adjustments. Colour font signifies the direction of change in the ACE-DD genotypes (red: down; green up).

Overall the results from the snapshot of metabolism post-exercise emphasize that compounds of anaplerotic reactions that replenish TCA cycle intermediates (i.e. aspartate and PEP [39]), were selectively reduced in ACE-DD genotypes after exercise (Fig 7). Concomitantly, compounds related to the removal of metabolic intermediates from the TCA cycle (cataplerosis) were less increased during recovery from exercise in ACE-DD genotypes (i.e. saccharopine, glutathione and PEP, S2 Table). TCA cycle intermediates were not detected with our UPLC-MS based measured. However they are known to be relatively stable and are unaffected during high-energy consumption with exercise, despite large alterations in carbon flux through the TCA cycle [40]. These findings, together with the observed indication for reduced aerobic energy metabolism in ACE-DD genotypes, are compatible with the idea that under the studied conditions the lack of the ACE I-allele is associated with a reduced oxidative carbon flux in exercised muscle resulting from an imbalance between anaplerosis and cataplerosis.

The identified differences in serum glucose concentration between ACE genotypes after the lab test were remarkably well confirmed by the pronounced rise in serum glucose after the field test. Conversely exercise-induced changes in serum levels of triglyceride were not affected by the ACE-I/D polymorphism (i.e. S1 Table). RER is an indicator of physical fitness [23]. Higher RER values are indicative of an increased glucose oxidation. Thus, our findings are indicative of a reduction in glucose oxidation in non-carriers of the ACE I-allele with one-legged exercise [21]. Because skeletal muscle is a main contributor to energy utilisation with exercise [41], our finding suggests that the elevated serum concentration of glucose in non-carriers of the ACE I-allele reflects a relatively reduced oxidation of glucose in contracting muscle. This is supported by a correlation between the changes in serum glucose and the average RER during the one-legged exercise. To our understanding, the connected changes identified here are the first to indicate that a modified muscle metabolism and glucose handling underlies the effect of the ACE-I/D polymorphism on the activity-induced metabolic phenotype in men [16,34]. Overall the present novel findings lead us to the interpretation that the lack of an ACE I-allele, in the studied subjects, is a disadvantageous condition for aerobic energy metabolism during and after endurance work which is compensated by a shift in energy production to metabolic alternatives of glucose oxidation.

Intriguingly, serum values of glucose in ACE-DD genotypes were high enough (mean of 7.9 mM) to technically meet the criteria of hyperglycaemia [42]. Serum glucose levels are tightly regulated in an interval between 4 and 7 mM through the regulation of the rate of glucose appearance and the rate of glucose disappearance. Elevations in serum glucose occur with the increase in hepatic glucose output such as after a meal. This can be excluded as explanation for our observation since subjects were fasted and angiotensin 2 does not regulate hepatic glucose [43]. By contrast the rate of glucose disappearance is affected by angiotensin 2/ACE through effects on insulin [19,22]. Given that hepatic glucose output is also increased with exercise [44] our findings of an ACE-I/D modulated response of pathways related to glucose handling with exercise (Fig 7) suggest a deficiency in the rate of glucose uptake in the largest organ of insulin action, skeletal muscle, which contributes up to 80% to whole body glucose disposal.

It has been pointed out before that the uptake of liver-derived glucose in contracting muscle is importantly increased within minutes after the onset of exercise [43] and depends on enhanced perfusion of contracting skeletal muscle [3,5]. Capillaries are a main effector for the vasodilatative action of insulin [45] and the degree of glucose tolerance is positively correlated with capillary density in skeletal muscle [46]. Therefore the rise in serum glucose with insulin resistance with type 2 diabetes appears to be mainly due to an incapacity for glucose uptake through the capillary endothelium [44]. The identified reduction in capillary-to-fibre ratio in *m*. *vastus lateralis* of ACE-DD genotypes; indicates that capillarisation is a possible limiting factor for glucose supply during endurance exercise in this genotype. A relationship between muscle metabolism and capillarisation is suggested from the correlations between capillary density and the changes in RER after the one-legged endurance exercise (r = 0.65). The investigation highlights that the suggested deficit in glucose metabolism with exhaustive exercise in the studied ACE-DD genotypes, as reflected by the accumulation of blood glucose, is related to a reduced capillarisation in the largest organ system of insulin action, skeletal muscle.

Our findings are of interest given the association of ACE-DD genotypes with a larger risk of developing type II diabetes [18,19]. Our findings indicate that this risk can be exposed in otherwise healthy subjects by assessing markers of glucose metabolism after an exhaustive test of exercise (especially the out-of norm elevation of glucose levels after exhaustive exercise). Our findings also build a connection between glucose handling and serum angiotensin 2 levels. For instance we identified a correlation between the fold changes in angiotensin 2 and muscle capillary density (r = 0. 65) and changes in glucose (r = –0.846; p = 0.008) after one-legged exercise. These latter observations are of interest given that the enzyme activity of ACE is regulated by shear stress acting on the endothelial lumen such a during exercise induced hyperemia [47] and because angiotensin 2 which increases in function of exercise intensity [10] affects arterio-venous glucose extraction and glucose utilisation in contracting muscle [21,48]. In our study serum angiotensin 2 concentrations were higher in ACE-DD genotypes at rest as predicted based on the effect of the presence of the ACE I-allele on the expression of ACE and the downstream processing of angiotensin 2 [10]. However, serum angiotensin 2 concentrations were not increased in ACE-DD genotypes after endurance exercise. We have recently identified that variability in the rise in serum angiotensin 2 after intense exercise and capillary perfusion is related to the ACE I/D polymorphism [10]. In this regard the overshoot of serum glucose post exercise in ACE-DD genotypes is of interest as these subjects may miss a vasodilatatory response post-exercise. Together, these relationships suggest that variability in serum glucose concentration with exhaustive muscle work is related to the capillary lumen that is serving the contracting musculature. Our findings imply that genetic effects of the ACE I-allele on serum angiotensin 2 levels modulate the former association. We thus interpret our findings in ACE-DD genotypes to reflect a reduced uptake of substrates for mitochondrial oxidation from the vascular compartment due to reduced capillarisation and elevated potential for vasoconstriction.

## Conclusions

Genetical influence of the upstream regulator of vasoconstrictor, ACE, in non-carriers of the ACE I-allele reduces endurance performance via a capillarisation-mediated reduction of glucose uptake or oxidation in working muscle. A number of major muscle metabolites are suggested as targets for future investigation apprehending the implicated myocellular processes.

## Supporting Information

S1 Fig Experimental design. Sketch of the experimental approach being pursued to asses the influence of the ACE-I/D gene polymorphism on system and local (i.e. muscle) metabolic variables of exercise through validating values between the lab and field test. Top, Venn diagram with the numbers of subjects in the different groups. Arrows signify effects, which were compared statistically.
(TIF)

S1 Table Serum metabolites after exhaustive one leg exercise. Median ± SE of the fold changes in metabolites in serum with the one-legged cycling exercise and respiration exchange ratio of the subjects, which completed the one-legged exercise. Abbreviations: LDL, low-density lipoprotein; HDL, high-density lipoprotein.
(DOCX)

S2 Table Muscle metabolome after exhaustive one leg exercise. Summary of the metabolites demonstrating altered levels 30 min after the one-legged cycling exercise in vastus lateralis muscle.
(DOCX)

S3 Table ACE-I/D genotype dependent response of muscle metabolites to exercise. List of post vs. pre exercise alterations for 19 identified metabolites, which demonstrated an ACE-I/D modulated response to one-legged endurance exercise. Metabolites which q-value for a genotype difference was below 0.1% are underlined.
(DOCX)

## References

[1] Horowitz JF, Klein S. Lipid metabolism during endurance exercise. Am J Clin Nutr. 2000;72: 558S–563S. 10919960 10.1093/ajcn/72.2.558S

[2] Roberts TJ, Weber JM, Hoppeler H, Weibel ER, Taylor CR. Design of the oxygen and substrate pathways. II. Defining the upper limits of carbohydrate and fat oxidation. J Exp Biol. 1996;199: 1651–1658. 8708572 10.1242/jeb.199.8.1651

[3] Rose AJ, Richter EA. Skeletal muscle glucose uptake during exercise: how is it regulated? Physiology. 2005;20: 260–270. 16024514 10.1152/physiol.00012.2005

[4] Essen-Gustavsson B, Blomstrand E. Effect of exercise on concentrations of free amino acids in pools of type I and type II fibres in human muscle with reduced glycogen stores. Acta Physiol Scand. 2002;174: 275–281. 11906327 10.1046/j.1365-201x.2002.00942.x

[5] Clifford PS, Hellsten Y. Vasodilatory mechanisms in contracting skeletal muscle. J Appl Physiol. 2004;97: 393–403. 15220322 10.1152/japplphysiol.00179.2004

[6] Korthuis RJ. Exercise Hyperaemia and Regulation of Tissue Oxygenation During Muscular Activity Skeletal Muscle Circulation. Morgan & Claypool Life Sciences, San Rafael (CA); 2011. 21850766

[7] Ahmed SK, Egginton S, Jakeman PM, Mannion AF, Ross HF. Is human skeletal muscle capillary supply modelled according to fibre size or fibre type? Exp Physiol. 1997;82: 231–234. 9023521 10.1113/expphysiol.1997.sp004012

[8] Carlson BE, Arciero JC, Secomb TW. Theoretical model of blood flow autoregulation: roles of myogenic, shear-dependent, and metabolic responses. Am J Physiol Heart Circ Physiol. 2008;295: H1572–1579. 10.1152/ajpheart.00262.2008 18723769 PMC2593503

[9] Halliwill JR. Mechanisms and clinical implications of post-exercise hypotension in humans. Exerc Sport Sci Rev. 2001;29: 65–70. 11337825 10.1097/00003677-200104000-00005

[10] van Ginkel S, de Haan A, Woerdeman J, Vanhees L, Serné E, Flück M, et al Exercise intensity modulates capillary perfusion in correspondence with ACE I/D modulated serum angiotensin II levels. Appl Trans Genomics. 2015;4: 33–37. 10.1016/j.atg.2015.03.002 PMC4745357 26937347

[11] Brothers RM, Haslund ML, Wray DW, Raven PB, Sander M. Exercise-induced inhibition of angiotensin II vasoconstriction in human thigh muscle. J Physiol. 2006;577: 727–737. 16973706 10.1113/jphysiol.2006.113977 PMC1890428

[12] Schwieler JH, Kahan T, Nussberger J, Hjemdahl P. Influence of the renin-angiotensin system on sympathetic neurotransmission in canine skeletal muscle in vivo. Naunyn Schmiedebergs Arch Pharmacol. 1991;343: 166–172. 1648672 10.1007/BF00168605

[13] Puthucheary Z, Skipworth JR, Rawal J, Loosemore M, Van Someren K, Montgomery HE. The ACE gene and human performance: 12 years on. Sports Med. 2011;41: 433–448. 10.2165/11588720-000000000-00000 21615186

[14] Rigat B, Hubert C, Alhenc-Gelas F, Cambien F, Corvol P, Soubrier F. An insertion/deletion polymorphism in the angiotensin I-converting enzyme gene accounting for half the variance of serum enzyme levels. J Clin Inves. 1990;86: 1343–1346. 10.1172/JCI114844 PMC296868 1976655

[15] van Ginkel S, Waldron S, Ruoss S, Rittweger J, Vaughan D, Flück M. COX4I2 expression post exercise is modified by Angiotensin converting enzyme 19th Annual ECSS-Congress. Amsterdam, the Netherlands; 2014. MO-PM54-7. Available: http://wp1191596.server-he.de/DATA/CONGRESSES/AMSTERDAM_2014/DOCUMENTS/AMSTERDAM_FinPro.pdf

[16] Vaughan D, Huber-Abel FA, Graber F, Hoppeler H, Fluck M. The angiotensin converting enzyme insertion/deletion polymorphism alters the response of muscle energy supply lines to exercise. Europ J Appl Physiol. 2013;113: 1719–1729. 10.1007/s00421-012-2583-6 PMC3677975 23397151

[17] Salem AH, Batzer MA. High frequency of the D allele of the angiotensin-converting enzyme gene in Arabic populations. BMC Res Notes. 2009;2: 99 10.1186/1756-0500-2-99 19505317 PMC2699340

[18] Panahloo A, Andres C, Mohamed-Ali V, Gould MM, Talmud P, Humphries SE, et al The insertion allele of the ACE gene I/D polymorphism. A candidate gene for insulin resistance? Circulation. 1995;92: 3390–3393. 8521557 10.1161/01.cir.92.12.3390

[19] Yang M, Qiu CC, Xu Q, Xiang HD (2006) Association of angiotensin converting enzyme gene I/D polymorphism with type 2 diabetes mellitus. Biomed Env Sci. 2006;19: 323–327. 17044652

[20] Staessen J, Fagard R, Hespel P, Lijnen P, Vanhees L, Amery A. Plasma renin system during exercise in normal men. J Appl Physiol. 1987;63: 188–194. 3305466 10.1152/jappl.1987.63.1.188

[21] Hurley BF, Nemeth PM, Martin WH 3rd, Hagberg JM, Dalsky GP, Holloszy JO. Muscle triglyceride utilization during exercise: effect of training. J Appl Physiol. 1986;60: 562–567. 3512511 10.1152/jappl.1986.60.2.562

[22] Huang XH, Rantalaiho V, Wirta O, Pasternack A, Koivula T, Hiltunen T, et al Relationship of the angiotensin-converting enzyme gene polymorphism to glucose intolerance, insulin resistance, and hypertension in NIDDM. Hum Genet 1998;102: 372–378. 9544854 10.1007/s004390050707

[23] Ramos-Jimenez A, Hernandez-Torres RP, Torres-Duran PV, Romero-Gonzalez J, Mascher D, Posadas-Romero C, et al The Respiratory Exchange Ratio is Associated with Fitness Indicators Both in Trained and Untrained Men: A Possible Application for People with Reduced Exercise Tolerance. Clin Med Circ Respirat Pulm Med. 2008;2: 1–9. 21157516 10.4137/ccrpm.s449 PMC2990231

[24] Harriss DJ, Atkinson G. Update—Ethical standards in sport and exercise science research. Int J Sports Med. 2011;32: 819–821. 10.1055/s-0031-1287829 22065312

[25] Ware JE Jr., Sherbourne CD. The MOS 36-item short-form health survey (SF-36). I. Conceptual framework and item selection. Medical Care. 1992;30: 473–483. 1593914

[26] Poole DC, Wilkerson DP, Jones AM. Validity of criteria for establishing maximal O2 uptake during ramp exercise tests. Europ J Appl Physiol. 2008;102: 403–410. 10.1007/s00421-007-0596-3 17968581

[27] van Ginkel S, Amami M, Dela F, Niederseer D, Narici MV, Flück M, et al Adjustments of muscle capillarity but not mitochondrial protein with skiing in the elderly. Scand J Med Sci Sports. 2015;25: e360–367. 10.1111/sms.12324 25262765

[28] Li R, Narici MV, Erskine RM, Seynnes OR, Rittweger J, Flück M, et al Costamere remodeling with muscle loading and unloading in healthy young men. J Anat. 2013;223: 525–536. 10.1111/joa.12101 24010829 PMC3916893

[29] Rifai N, Warnick GR, McNamara JR, Belcher JD, Grinstead GF, Frantz ID Jr. Measurement of low-density-lipoprotein cholesterol in serum: a status report. Clin Chem. 1992;38: 150–160. 1733589

[30] Buescher JM, Moco S, Sauer U, Zamboni N. Ultrahigh performance liquid chromatography-tandem mass spectrometry method for fast and robust quantification of anionic and aromatic metabolites. Anal Chem. 2010;82: 4403–4412. 10.1021/ac100101d 20433152

[31] Matyash V, Liebisch G, Kurzchalia TV, Shevchenko A, Schwudke D. Lipid extraction by methyl-tert-butyl ether for high-throughput lipidomics. J Lipid Res. 2008;49: 1137–1146. 10.1194/jlr.D700041-JLR200 18281723 PMC2311442

[32] Tusher VG, Tibshirani R, Chu G. Significance analysis of microarrays applied to the ionizing radiation response. Proceedings of the Natl Acad Sci USA. 2001;98: 5116–5121. 10.1073/pnas.091062498 PMC33173 11309499

[33] Williams AG, Rayson MP, Jubb M, World M, Woods DR, Hayward M, et al The ACE gene and muscle performance. Nature. 2000;403: 614. 10.1038/35001141 10688186

[34] Flueck M, Vaughan D, Westerblad H. Linking genes with exercise: where is the cut-off? Eur J Appl Physiol. 2010;110: 1095–1098. 10.1007/s00421-010-1662-9 20941629

[35] Myerson S, Hemingway H, Budget R, Martin J, Humphries S, Montgomery H. Human angiotensin I-converting enzyme gene and endurance performance. J Appl Physiol. 1999;87: 1313–1316. 10517757 10.1152/jappl.1999.87.4.1313

[36] McPhee JS, Williams AG, Stewart C, Baar K, Schindler JP, et al The training stimulus experienced by the leg muscles during cycling in humans. Exp Physiol. 2009;94: 684–694. 10.1113/expphysiol.2008.045658 19218358

[37] Dunn WB, Broadhurst D, Begley P, Zelena E, Francis-McIntyre S, Anderson N, et al Procedures for large-scale metabolic profiling of serum and plasma using gas chromatography and liquid chromatography coupled to mass spectrometry. Nature protocols 2011;6: 1060–1083. 10.1038/nprot.2011.335 21720319

[38] Romijn JA, Coyle EF, Sidossis LS, Gastaldelli A, Horowitz JF, Endert E et al Regulation of endogenous fat and carbohydrate metabolism in relation to exercise intensity and duration. Am J Physiol 1993;265: E380–391. 8214047 10.1152/ajpendo.1993.265.3.E380

[39] Owen OE, Kalhan SC, Hanson RW. The key role of anaplerosis and cataplerosis for citric acid cycle function. J Biol Chem. 2002;277: 30409–30412. 12087111 10.1074/jbc.R200006200

[40] Graham TE, Gibala MJ. in Skeletal Muscle Metabolism in Exercise and Diabetes New York: Plenum Press; 1998 pp. 271–286.

[41] Darveau CA, Suarez RK, Andrews RD, Hochachka PW. Allometric cascade as a unifying principle of body mass effects on metabolism. Nature. 2002;417: 166–170. 12000958 10.1038/417166a

[42] Association' AD. Diagnosis and classification of diabetes mellitus. Diabetes Care 2010;33 S1: S62–69. 20042775 10.2337/dc10-S062 PMC2797383

[43] Bergeron R, Kjaer M, Simonsen L, Bulow J, Skovgaard D, Howlett K, et al Splanchnic blood flow and hepatic glucose production in exercising humans: role of renin-angiotensin system. Am J Physiol Reg Int Comp Physiol. 2001;81: R1854–1861. 10.1152/ajpregu.2001.281.6.R1854 11705770

[44] Bird SR, Hawley JA. Exercise and type 2 diabetes: new prescription for an old problem. Maturitas. 2012;72: 311–316. 10.1016/j.maturitas.2012.05.015 22748760

[45] Sowers JR. Insulin and insulin-like growth factor in normal and pathological cardiovascular physiology. Hypertension 1997;29: 691–699. 9052883 10.1161/01.hyp.29.3.691

[46] Lithell H, Lindgarde F, Hellsing K, Lundqvist G, Nygaard E, Vessby B, et al Body weight, skeletal muscle morphology, and enzyme activities in relation to fasting serum insulin concentration and glucose tolerance in 48-year-old men. Diabetes. 1981;30: 19–25. 10.2337/diab.30.1.19 7014301

[47] Rieder MJ, Carmona R, Krieger JE, Pritchard KA, Jr., Greene AS. Suppression of angiotensin-converting enzyme expression and activity by shear stress. Circ Res. 1997;80: 312–319. 9048650 10.1161/01.res.80.3.312

[48] Jamerson KA, Nesbitt SD, Amerena JV, Grant E, Julius S. Angiotensin mediates forearm glucose uptake by hemodynamic rather than direct effects. Hypertension 1996;27: 854–858. 8613260 10.1161/01.hyp.27.4.854

