# Supplementary figures and images for: The Angiotensin Converting Enzyme Insertion/Deletion Polymorphism Modifies Exercise-Induced Muscle Metabolism
Source: PLoS One. 2016 Mar 16;11(3):e0149046. doi: 10.1371/journal.pone.0149046 (PMC4794249; doi:10.1371/journal.pone.0149046)

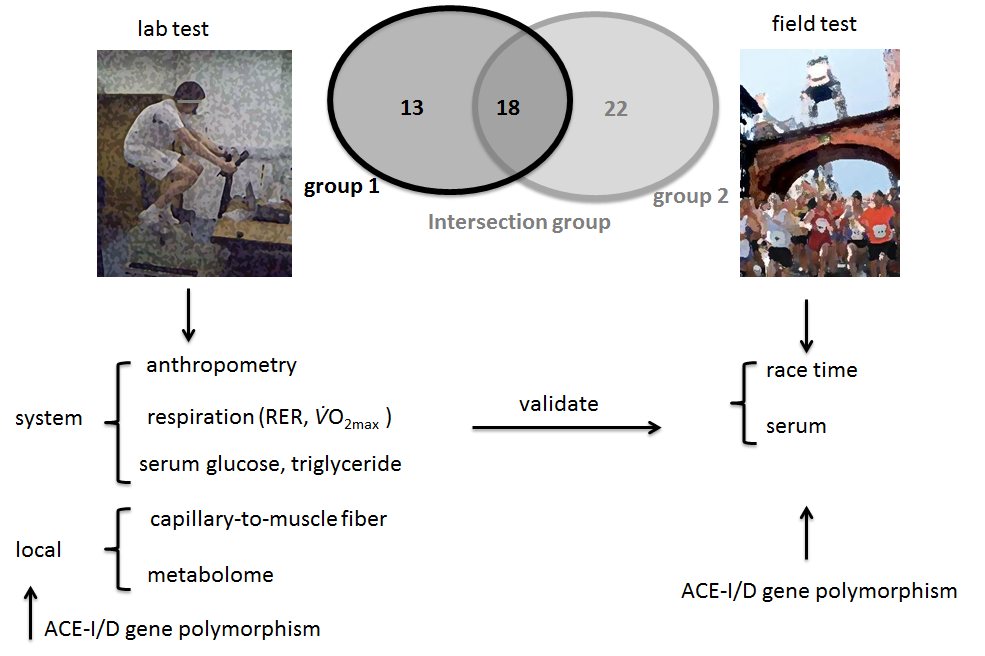

Supplement: S1 Fig — Sketch of the experimental approach being pursued to asses the influence of the ACE-I/D gene polymorphism on system and local (i.e. muscle) metabolic variables of exercise through validating values between the lab and field test. Top, Venn diagram with the numbers of subjects in the different groups. Arrows signify effects, which were compared statistically. (TIF) [file pone.0149046.s001.tif]
