# Supplementary material for: The Angiotensin Converting Enzyme Insertion/Deletion Polymorphism Modifies Exercise-Induced Muscle Metabolism
Source: PLoS One. 2016 Mar 16;11(3):e0149046. doi: 10.1371/journal.pone.0149046 (PMC4794249; doi:10.1371/journal.pone.0149046)
Supplement: S1 Table — Median ± SE of the fold changes in metabolites in serum with the one-legged cycling exercise and respiration exchange ratio of the subjects, which completed the one-legged exercise. Abbreviations: LDL, low-density lipoprotein; HDL, high-density lipoprotein. (DOCX) [file pone.0149046.s002.docx]

***S1 table:* Serum metabolites after exhaustive one leg exercise**

Median ± SE of the fold changes in metabolites in serum with the one-legged cycling exercise and respiration exchange ratio of the subjects, which completed the one-legged exercise. Abbreviations: LDL, low density lipoprotein; HDL, high density lipoprotein.

**n Glucose Cholesterol LDL Triglycerides Ketones HDL RER**

[mmol] [mmol] [mmol] [mmol] [mmol] [mmol**] [CO2/O2]**

ACE-DD 11 1.37 ± 0.06 1.04 ± 0.03 1.00 ± 0.05 1.00 ± 0.11 0.98 ± 0.08 1.10 ± 0.25 1.19 ± 0.03

ACE-ID 10 1.08 ± 0.06 1.04 ± 0.05 0.96 ± 0.09 0.84 ± 0.09 0.88 ± 0.11 1.36 ± 0.23 1.12 ± 0.04

ACE-II 7 1.07 ± 0.05 1.04 ± 0.04 1.02 ± 0.07 0.94 ± 0.10 1.06 ± 0.09 1.05 ± 0.05 1.18 ± 0.04

DD vs. ID/II <0.001 0.92 0.84 0.45 0.35 0.71 0.87

ACE-DD vs II 0.004 0.93 0.98 1.00 0.85 0.69 0.43
