# Supplementary material for: The Angiotensin Converting Enzyme Insertion/Deletion Polymorphism Modifies Exercise-Induced Muscle Metabolism
Source: PLoS One. 2016 Mar 16;11(3):e0149046. doi: 10.1371/journal.pone.0149046 (PMC4794249; doi:10.1371/journal.pone.0149046)
Supplement: S2 Table — Summary of the metabolites demonstrating altered levels 30 min after the one-legged cycling exercise in vastus lateralis muscle. (DOCX) [file pone.0149046.s003.docx]

***S2 table:* Muscle metabolome after exhaustive one leg exercise.** Summary of the metabolites demonstrating altered levels 30 min after the one-legged cycling exercise in vastus lateralis muscle.

**name formula neutral mass (Da) retention m/z compound post vs. pre q-value (%) function/ ontology**

2-Keto-glutaramic acid C5H7NO4 145.0376366 6.95 144.03067 HMDB01552 3.2 0.0 deaminated metabolite of glutamine

Saccharopine C11H20N2O6 276.1343693 8.25 275.12709 HMDB00279 5.8 0.0 degradation of lysine.

Glutathione C10H17N3O6S 307.0848372 7.89 306.07756 HMDB00125 3.1 0.0 synthesized from cysteine

sopropyl β-D-glucoside C9H18O6 222.1115493 7.66 203.09371 HMDB32705 2.0 0.0 found in herbs and spices

cis-Piceid C20H22O8 390.1360138 7.1 427.08438 HMDB31422 21.2 0.0 constituent of the wine grape

Axillarenic acid C24H46O4 398.340 11.51 416.374 123060238 3.1 0.0 fatty Acyls

Dichotellate A C26H42O4 418.309 11.56 419.316 123060263 0.8 0.0 fatty Acyls

1,3Z,6Z,9Z-Heneicosatetraene C21H36 288.281 13.17 352.297 135637230 0.6 0.0 fatty Acyls

2,6-Dimethyl-1,8-octanedioic acid C10H18O4 202.123 7.57 225.112 135636040 2.3 0.0 fatty Acyls

3,11-dihydroxy myristoic acid C14H28O4 260.199 8.47 225.185 7850178 0.8 0.0 fatty Acyls

5E-Dodecenyl acetate C14H26O2 226.194 8.63 244.227 135636564 0.6 0.0 fatty Acyls

Type IV cyanolipid eicosanoyl ester C25H45NO2 391.345 8.65 374.342 49703481 139286.4 0.0 fatty Acyls

Syzygiol C18H18O5 314.116 8.1 332.149 74381027 2489.9 0.9 polyketides

(4R,6R)-cis-Carveol C10H16O 152.120 8.63 153.128 74382177 0.7 0.0 prenol Lipids

Sugeonyl acetate C17H24O3 276.173 8.68 294.207 123067406 1.2 0.9 prenol Lipids

(4E,8E,d18:2) sphingosine C18H35NO2 297.267 10.58 298.275 74382659 4.3 0.0 sphingolipids

Prosopinine C16H33NO3 287.247 8.84 288.254 74382698 0.8 0.0 sphingolipids

3beta-Hydroxy-7-oxo-5alpha- C24H38O4 390.278 11.37 391.285 7851039 0.8 0.0 sterol Lipids

cholan-24-oic Acid
