# Supplementary material for: The Angiotensin Converting Enzyme Insertion/Deletion Polymorphism Modifies Exercise-Induced Muscle Metabolism
Source: PLoS One. 2016 Mar 16;11(3):e0149046. doi: 10.1371/journal.pone.0149046 (PMC4794249; doi:10.1371/journal.pone.0149046)
Supplement: S3 Table — List of post vs. pre exercise alterations for 19 identified metabolites, which demonstrated an ACE-I/D modulated response to one-legged endurance exercise. Metabolites which q-value for a genotype difference was below 0.1% are underlined. (DOCX) [file pone.0149046.s004.docx]

***S3 table:* ACE-I/D genotype dependent response of muscle metabolites to exercise.** List of post vs. pre exercise alterations for 19 identified metabolites, which demonstrated an ACE-I/D modulated response to one-legged endurance exercise. Metabolites which q-value for a genotype difference was below 0.1% are underlined.

**Description Formula compound ACE-DD ACE-ID ACE-II DD v ID DD V II D vs no D function/ ontology**

NADP+ C21H29N7O17P3 HMDB00217 0.5 1.1 1.5 0.4 0.3 0.4 pyruvate metabolism

Phosphoenolpyruvic C3H5O6P HMDB00263 0.5 1.1 1.0 0.5 0.5 0.5 pyruvate metabolism

acid

Methylhippuric acid C10H11NO3 HMDB00859 0.6 2.3 6.1 0.3 0.1 0.2 disorders of β-oxidation

Glutathione C10H17N3O6S HMDB00125 2.0 4.0 5.2 0.5 0.4 0.5 glutathione metabolism

2-Bromoacetaldehyde C2H3BrO HMDB60344 81.0 9.6 54.3 8.4 1.5 3.9 glutathione metabolism

L-Aspartic acid C4H7NO4 HMDB00191 0.6 9.1 10.8 0.1 0.1 0.1 amino acid metabolism

Saccharopine C11H20N2O6 HMDB00279 3.8 6.5 10.8 0.6 0.4 0.5 amino acid metabolism

Isopentyl beta-D- C11H22O6 HMDB34750 0.5 1.3 1.7 0.4 0.3 0.3 food

glucoside

3'-Glucosyl-2',4',6'-tri C14H18O9 HMDB40621 0.4 0.7 1.3 0.6 0.3 0.5 food

hydroxyacetophenone

cis-Piceid C20H22O8 HMDB31422 32.7 14.1 8.1 2.3 4.0 2.6 food

Garcinia acid C6H8O8 HMDB31159 0.4 1.3 2.2 0.3 0.2 0.3 food

Carbadox C11H10N4O4 HMDB31762 3.6 824.7 3.9 0.0 0.9 0.0 food

Sulfacytine C12H14N4O3S HMDB15412 10.5 23.8 13.5 0.4 0.8 0.5 medication

Ranitidine C13H22N4O3S HMDB01930 0.6 1.1 1.5 0.5 0.4 0.5 medication

Amlexanox C16H14N2O4 HMDB15160 1.4 2.1 1.8 0.6 0.7 0.7 medication

Idarubicin C26H27NO9 HMDB15308 0.4 1.1 1.5 0.4 0.3 0.4 medication

Clofazimine C27H22Cl2N4 HMDB14983 0.9 0.9 0.4 1.1 2.2 1.2 medication

Astemizole C28H31FN4O HMDB14775 0.8 0.7 0.5 1.1 1.7 1.2 medication

4-Hydroxyclonidine C9H9Cl2N3O HMDB60555 2.3 1.6 0.8 1.5 2.8 1.7 medication
